# FTD Disorders Registry (FTDDR) Participants: Understanding Social Risk Factors in Research Participation

**DOI:** 10.1002/alz.089807

**Published:** 2025-01-09

**Authors:** Sweatha Reddy, Robert Reinecker, Carrie Milliard, Hilary W. Heuer, Leah K. Forsberg, Adam L. Boxer, Howard J. Rosen, Brad F. Boeve

**Affiliations:** ^1^ FTD Disorders Registry, King of Prussia, PA USA; ^2^ University of California San Francisco (UCSF), San Francisco, CA USA; ^3^ Department of Neurology, Mayo Clinic, Rochester, MN USA; ^4^ Memory and Aging Center, UCSF Weill Institute for Neurosciences, San Francisco, CA USA; ^5^ UCSF Weill Institute for Neurosciences, University of California, San Francisco, CA USA

## Abstract

**Background:**

Frontotemporal degeneration (FTD) is an umbrella term encompassing a range of rare neurodegenerative disorders that cause progressive changes to behavior, personality, language, and movement with onset typically before age 60. Currently, several potential FTD therapies are under investigation, underscoring the need for increased diversity in research participation. Two validated scores describe socioeconomic and geographic factors that may impact willingness to participate in research. The Area Deprivation Index (ADI) assesses socioeconomic disadvantages and captures social determinants of health. The Rural‐Urban Commuting Area Codes (RUCA) measures population density, urbanization, and daily commuting to determine individuals living in urban versus rural communities. FTDDR is a compliant, web‐based registry to facilitate FTD research. ADI and RUCA analyses of FTDDR participants can provide insight into current participants’ demographics and areas for recruitment prioritization.

**Method:**

FTDDR participants living in the United States were divided into two cohorts: (1‐FTDDR) enrolled in FTDDR only and (2‐ALLFTD) enrolled in FTDDR through ALLFTD, a longitudinal natural history study. ADI was calculated from self‐reported zip codes for 1392 participants in cohort 1 and 983 participants in cohort 2. RUCA scores were generated using self‐reported zip codes for 4565 participants from cohort 1 and 1174 participants from cohort 2.

**Result:**

ADI analysis showed a low percentage (6.9% FTDDR, 5.7% ALLFTD) of participants lived in highly disadvantageous communities. 40.5% of FTDDR cohort and 49.3% of ALLFTD cohort lived in communities considered “low disadvantage,” suggesting they may have fewer socioeconomic risk factors or impediments to research participation. Most participants in both cohorts lived in urban areas (87.9% FTDDR, 85.8% ALLFD); only 3.2% of both cohorts lived in rural communities.

**Conclusion:**

Socioeconomic and geographic factors may play a role in accurate diagnosis, access to medical centers, and ability to participate in research. ADI and RUCA analyses indicate that the majority of FTDDR participants, including those actively enrolled in FTD research, live in less disadvantageous neighborhoods and in urban areas, highlighting the need for outreach to more disadvantaged and rural communities. FTDDR prioritizing an engagement strategy to identify, recruit, and retain diverse individuals willing to participate in research could accelerate advancements in FTD research.

